# Supplementary material for: Factors influencing walking trips. Evidence from Gdynia, Poland
Source: PLoS One. 2021 Aug 4;16(8):e0254949. doi: 10.1371/journal.pone.0254949 (PMC8336852; doi:10.1371/journal.pone.0254949)
Supplement: S1 File — (DOC) [file pone.0254949.s001.doc]

**Study of pedestrian traffic in Downtown Gdynia - XI/XII.2017** *[translation from Polish language]*

We would like to ask you to fill in the questionnaire concerning pedestrian traffic in Downtown Gdynia. The survey is a part, along with the measurement of pedestrian traffic volume, of a comprehensive traffic study carried out within the project "Furthering Less Congestion by creating Opportunities for more Walking and cycling - FLOW". The survey is anonymous.

1. **Where are you coming from (street, building no.)**

……………………………………………………………………………….

1. **What’s your origin of travel?** */one answer/*

| House | Work |
| --- | --- |
| School | Healthcare |
| Public institutions | Shopping |
| Business meeting | Eating |
| Walking | Other: ………………………………………….. |

1. **Where are you going to?** (street, building no.)

……………………………………………………………………………….

1. **What’s your destination of travel?** */one answer /*

| House | Work |
| --- | --- |
| School | Healthcare |
| Public institutions | Shopping |
| Business meeting | Eating |
| Walking | Other: ………………………………………….. |

1. **How much time does the today’s walking trip take?**

| Less than 10 minutes | 10 – 30 minutes |
| --- | --- |
| More than 30 minutes | I don’t know |

1. **How did you reach the place where your walking trip started?**

| By car | Public transport |
| --- | --- |
| By bike | Other: ………………………………………….. |
| Doesn’t apply [when the respondent has left house on foot] | |

1. **What’s your view of the following aspects of walking in the centre of Gdynia?**

| ----- | POOR | FAIR | AVERAGE | GOOD | EXCELLENT |
| --- | --- | --- | --- | --- | --- |
| PERSONAL SAFETY IN GENERAL |  |  |  |  |  |
| ROAD SAFETY |  |  |  |  |  |
| MOVEMENT COMFORT |  |  |  |  |  |
| SERVICE ACCESSIBILITY |  |  |  |  |  |
| PUBLIC TRANSPORT ACCESSIBILITY |  |  |  |  |  |

1. **What’s your view on the availability (number of) the following facilities in the centre of Gdynia?**

| ----- | POOR | FAIR | AVERAGE | GOOD | EXCELLENT |
| --- | --- | --- | --- | --- | --- |
| BENCHES |  |  |  |  |  |
| GREEN AREAS |  |  |  |  |  |
| TREES AND BUSHES |  |  |  |  |  |

1. **Do you think there are streets in the centre of Gdynia in which the walking accessibility could be improved?**

| Yes | No |
| --- | --- |

*If yes, than where: ……………………..…………………………………………………………………………………………...*

1. **How do you perceive the pedestrian crossings in the centre of Gdynia?**

| ----- | POOR | FAIR | AVERAGE | GOOD | EXCELLENT |
| --- | --- | --- | --- | --- | --- |
| PEDESTRIAN CROSSING WAITING TIME |  |  |  |  |  |
| PEDESTRIAN CROSSING AVAILABILITY |  |  |  |  |  |
| DURATION OF GREEN LIGHT AT CROSSINGS |  |  |  |  |  |
| ELEVATED PEDESTRIAN CROSSING |  |  |  |  |  |
| PEDESTRIAN CROSSING SAFETY |  |  |  |  |  |
| TRAFFIC LIGHTS |  |  |  |  |  |

*What’s the pedestrian crossing which could be improved the most? …………………………………………………………………….………………………………………………...*

1. **Do you think in the centre of Gdynia more facilities for the people with moving limitations could be improved?**

| Yes | No |
| --- | --- |

*If yes, than where: ……………………..…………………………………………………………………………………………...*

1. **Are there any places on this very street where you stop and spend more time?**

| Yes | No |
| --- | --- |

*If yes, then what are those places and why do you spend more time there?: …..………………………………………………………………………………………………………………………………………………*

1. **Would you spend more time on this street if the following solutions were introduced:**

| - Only pedestrian travel allowed   Yes  No  Doesn’t matter |
| --- |
| - Only pedestrian and bike travel allowed   Yes  No  Doesn’t matter |
| - Only pedestrian and bike travel and public transport allowed   Yes  No  Doesn’t matter |
| - A zone of limited parking   Yes  No  Doesn’t matter |

1. **What’s your assessment of the pedestrian needs on this very street?**

| ----- | POOR | FAIR | AVERAGE | GOOD | EXCELLENT |
| --- | --- | --- | --- | --- | --- |
| PAVEMENT SURFACE |  |  |  |  |  |
| PAVEMENT WIDTH |  |  |  |  |  |
| PERSONAL SAFETY AT PAVEMENTS |  |  |  |  |  |
| EASINESS OF ROAD CROSSING |  |  |  |  |  |
| PEDESTRIAN SAFETY AT THE ROAD |  |  |  |  |  |
| ROAD AESTHETICS |  |  |  |  |  |
| LEISURE ACTIVITIES |  |  |  |  |  |

1. **Do you have any additional remarks regarding the walkability in the centre of Gdynia?**

………………………………………………………………………………………………………………………………………………………………………………………………………………………………………………………………………………………………………………………………………………………………………………………………………………………………………………………………

**15. Gender**

| Female | Male | Other |
| --- | --- | --- |

1. **Age**

| Less than 13 years | 14-18 | 19-24 |
| --- | --- | --- |
| 25-34 | 35-44 | 45-54 |
| 55-64 | 65-75 | More than 75 years |

1. **How many children do you have?**

| 0 | 1 |
| --- | --- |
| 2 | 3 and more |

1. **How many cars are there in your household?**

| 0 | 1 |
| --- | --- |
| 2 | 3 and more |

1. **What’s your economic status?**

| Employed | Student |
| --- | --- |
| Student and employed | Unemployed |
| Maternal/paternal leave | Pensioner |
| Other: ………………………………………….. |  |

1. **What’s your place of residence?**

……………………………………………………………………………….

1. **Where do you work/study?**

……………………………………………………………………………….

1. **How do you travel most often?**

| Car | Public transport |
| --- | --- |
| Bike | On foot |
| Car + public transport | Other: ………………………………………….. |

**Interviewer**

| Date: ………………………………… | Nr of the interviewing point: …………… |
| --- | --- |
| Wind: lack / light / moderate / strong | Cloudiness: lack / moderate / total |
| Rainfall: yes / no | Temperature: ………………. |
